# Molecular Mechanisms of the Protective Effects of Olive Leaf Polyphenols against Alzheimer’s Disease

**DOI:** 10.3390/ijms24054353

**Published:** 2023-02-22

**Authors:** Jose M. Romero-Márquez, Tamara Y. Forbes-Hernández, María D. Navarro-Hortal, Rosa Quirantes-Piné, Giuseppe Grosso, Francesca Giampieri, Vivian Lipari, Cristina Sánchez-González, Maurizio Battino, José L. Quiles

**Affiliations:** 1 Department of Physiology, Institute of Nutrition and Food Technology “José Mataix Verdú”, Biomedical Research Centre, University of Granada, 18016 Armilla, Spain; 2 Research and Development Functional Food Centre (CIDAF), Health Science Technological Park, Avenida del Conocimiento 37, 18016 Granada, Spain; 3 Department of Biomedical and Biotechnological Sciences, University of Catania, 95123 Catania, Italy; 4 Center for Human Nutrition and Mediterranean Foods (NUTREA), University of Catania, 95123 Catania, Italy; 5 Research Group on Foods, Nutritional Biochemistry and Health, Universidad Europea del Atlántico, Isabel Torres 21, 39011 Santander, Spain; 6 Department of Project Management, Universidad Internacional Iberoamericana, Campeche 24560, Mexico; 7 Department of Prohect Management, Universidade Internacional do Cuanza, Cuito 250, Bié, Angola; 8 Sport and Health Research Centre, University of Granada, C/Menéndez Pelayo 32, 18016 Granada, Spain; 9 Department of Clinical Sciences, Polytechnic University of Marche, 60131 Ancona, Italy; 10 International Joint Research Laboratory of Intelligent Agriculture and Agri-Products Processing, Jiangsu University, Zhenjiang 212013, China

**Keywords:** olive leaves, bioactive compounds, Alzheimer’s Disease, oleuropein, hydroxytyrosol

## Abstract

Alzheimer’s Disease (AD) is the cause of around 60–70% of global cases of dementia and approximately 50 million people have been reported to suffer this disease worldwide. The leaves of olive trees (*Olea europaea*) are the most abundant by-products of the olive grove industry. These by-products have been highlighted due to the wide variety of bioactive compounds such as oleuropein (OLE) and hydroxytyrosol (HT) with demonstrated medicinal properties to fight AD. In particular, the olive leaf (OL), OLE, and HT reduced not only amyloid-β formation but also neurofibrillary tangles formation through amyloid protein precursor processing modulation. Although the isolated olive phytochemicals exerted lower cholinesterase inhibitory activity, OL demonstrated high inhibitory activity in the cholinergic tests evaluated. The mechanisms underlying these protective effects may be associated with decreased neuroinflammation and oxidative stress via NF-κB and Nrf2 modulation, respectively. Despite the limited research, evidence indicates that OL consumption promotes autophagy and restores loss of proteostasis, which was reflected in lower toxic protein aggregation in AD models. Therefore, olive phytochemicals may be a promising tool as an adjuvant in the treatment of AD.

## 1. Introduction

Alzheimer’s Disease (AD) is the cause of around 60–70% of global cases of dementia [1] and approximately 50 million people have been reported to suffer from this disease worldwide [2]. In fact, AD incidence rates double every 5 years from 60 years of age [3] and it is estimated that dementia will affect 81.1 million people worldwide in 2040 [2,4].

The etiopathogenesis of AD is characterized by two histopathological events: the senile plaque aggregation formed by amyloid-β peptides (Aβ) in the central nervous system and the formation of neurofibrillary tangles (NFTs) associated with the accumulation of Tau protein in the hippocampus, neocortical area, and amygdala [4]. These events are associated with an increase in mitochondrial dysfunction, oxidative stress, glucose homeostasis alteration in the brain, neuroinflammation, and disturbances in the proteostatic network, which favor the appearance of senile plaques and NFTs, generating atrophy and neuron death characteristic of AD [5].

AD is a multifactorial disease whose appearance and development are marked by the interaction between genetic predisposition and external factors throughout life [4]. Among the risk factors, aging, gender (higher incidence in women), alcohol and tobacco consumption, obesity, and metabolic disorders such as diabetes mellitus, as well as a low cultural level and family history, have been highlighted [1]. Some of these risk factors, including physical activity, diet, smoking, and alcoholism, could be modified in order to reduce the onset of the disease [4].

Although there is still no pharmacological therapy for its treatment, the preventive and/or therapeutic nutritional interventions against AD have been gaining prominence in recent years [1]. It is known that 35% of dementias could be caused by modifiable risk factors associated with lifestyle, including the type of diet [1]. In particular, the Mediterranean Diet (MD), characterized by a high consumption of legumes, vegetables, fruits, vitamins, and virgin olive oil, and a low consumption of red meat, has been shown to reduce the incidence of AD [1]. MD presents a high contribution of bioactive substances such as phenolic compounds, which have been shown to exert a protective effect in AD [6]. In particular, the intake of phytochemical compounds naturally present in foods, such as oleuropein (OLE), hydroxytyrosol (HT), luteolin (LU), catechin, and curcumin, are related to neuroprotective effects in AD through the modulation of mechanisms such as oxidative stress and neuroinflammation, besides reducing the deposition and toxicity of the misfolded proteins involved [7,8,9,10,11,12].

The leaves of the olive tree (*Olea europaea*) are the most abundant by-product in the olive grove industry. In Spain, between 1 and 5 tons of waste are generated per hectare in the form of branches and leaves. OLs are long, hard, and lanceolate, and their edges curl up due to desiccation [13]. It is possible to obtain olive leaf extracts enriched in certain compounds after grinding and processing them with an extraction solvent such as methanol, ethanol, or water, or a mixture thereof. In addition, separation or concentration procedures can be applied to enrich extracts in particular molecules [14]. OLs have excellent medicinal properties thanks to the wide variety of bioactive compounds present in them. In this review, the preventive and therapeutic effect of olive compounds against AD were reviewed from the point of view of the molecular mechanisms involved.

## 2. Phytochemical Characterization of Olive Leaves

**Figure 1:**
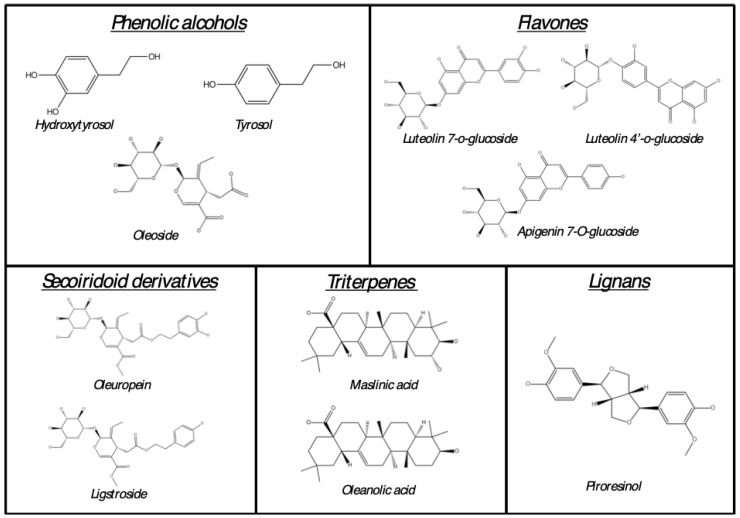
Most representative phytochemical compounds in OL.

OLs are consumed worldwide as a nutraceutical product due to their numerous and demonstrated health properties. In fact, considerable attention has been given to OLs because of their remarkable content of polyphenols [15]. The most representative compounds of OLs are those illustrated in Figure 1. Table 1 shows most representative compounds present in olive leaves. To date, the best-known phenolic compounds in OLs are secoiridoid derivatives, of which OLE is the most abundant. Additionally, the presence of phenolic alcohols (e.g., HT, tyrosol, and oleoside) and flavones (e.g., LU and luteolin-7-o-glucoside) should be highlighted together with others.

In the same way, other phenolic compounds, such as phenolic acids (e.g., verbascoside), flavanols (e.g., Epicatechin gallate), and flavonols (e.g., kaempferol-7-O-glucoside and rutin), have also been described in OLs (Figure 1). The phytochemical profile of the OL can be affected by several factors, such as the olive tree’s geographical location, cultivars, harvest season, drying temperature of leaves, and the solvents used for extraction. In this regard, Kabbash et al. (2021) demonstrated that olive leaves from Spain presented higher total flavonoid, phenolic, and OLE content compared with olive leaves from Italy and Egypt [16]. Similar results were obtained by Zhang et al. (2022), which showed a higher total flavonoid, OLE, and HT content, among other phenolic compounds, in Spanish olive leaves compared with those harvested in China or Italy [17]. These differences could also be attributed to the use of different cultivars. In this context, Nicoli et al. (2019) found significant differences among 15 different olive tree cultivars from Italy regarding OLE, HT, verbascoside (VB), and flavones (LU and luteolin-7-O-glucoside) content [18]. In the same way, Pasković et al. (2022) reported several differences in OLE, VB, rutin, catechin, phenolic alcohol (HT and tyrosol), and flavone (LU and apigenin derivatives) content in four different olive tree cultivars from Croatia [19]. Furthermore, the harvesting season has been demonstrated to influence the content of most of the phenolic compounds of OLs, even those of the same cultivar, with increases in olives harvested between March and April [16,19]. Additionally, it has been reported that the temperature of leaf drying after pruning can also affect the phenolic content. In this context, olive leaves dried using a freezing protocol (−80 °C) presented higher amounts of phytochemicals compared with those dried using a hot air protocol (105 °C), with the exception of OLE, the content of which increased in hot-air-dried olive leaves [17]. Authors attributed these results to the fact that some molecules, such as flavonoids, easily break down into smaller compounds during dehydration, while the stability of secoiroids, such as OLE, is relatively high. Finally, the method and solvent used for the extraction of olive leaf polyphenols has been reported to dramatically influence the final phenolic content. Most of the evaluated studies in this work used different proportions of methanol [19,20,21,22,23], ethanol [16,17,18], or water [23,24] as solvent to obtain the olive leaf extract. However, only Kontogianni et al. (2013) evaluated the direct influence of the solvent used in OL polyphenol extraction. Independently of the detection method (high performance liquid chromatography or nuclear magnetic resonance spectroscopy), the olive leaves extracted with methanol presented a higher content of OLE, HT, and LU and its derivatives compared with the aqueous OL extract. In fact, LU was not detected in the aqueous extract [23]. For further studies, the phytochemical characterization of OL needs to be standardized to generate a homogeneous phenolic profile through the regulation of the previously mentioned factors.

**Table 1:** Phytochemical characterization of olive leaves.

Phenolic Compound	CAS Number	Molecular Formula	Range of Content	Refs.
**Phenolic acids**				
Verbascoside	61276-17-3	C_29_H_36_O_15_	9–465.63	[18,19,20,22,24,25,26]
Gallic acid	149-91-7	C_7_H_6_O_5_	0.17–140	[22,24,25]
Vanillin	121-33-5	C_8_H_8_O_3_	nd–0.05	[24,25]
Vanillic acid	121-34-6	C_8_H_8_O_4_	3–274	[20,22,24]
p-coumaric acid	501-98-4	C_9_H_8_O_3_	nd–4.41	[20,24,25]
Ferulic acid	537-98-4	C_10_H_10_O_4_	nd–0.96	[24,25]
Chlorogenic acid	327-97-9	C_16_H_18_O_9_	nd–26	[17,20,22,24,25]
Neochlorogenic acid	906-33-2	C_16_H_18_O_9_	25	[22]
Caffeic acid	331-39-5	C_9_H_8_O_4_	nd–20.15	[20,24,25]
Plantamajoside	104777-68-6	C_29_H_36_O_16_	nd–1.35	[17]
Rosmarinic acid	20283-92-5	C18H16O8	0.59	[24]
**Phenolic alcohols**				
Hydroxytyrosol	10597-60-1	C8H10O3	6.68–1092.74	[17,19,23,25]
Hydroxytyrosol 4-O-glucoside	54695-80-6	C_14_H_20_O_8_	nd–11.02	[17]
p-Hydroxybenzoic acid	99-96-7	C7H6O3	3.63–141	[20,22,24]
2,3-Dihydroxybenzoic acid	303-38-8	C7H6O4	2	[22]
2,5-Dihydroxybenzoic acid	490-79-9	C7H6O4	1.18	[24]
3,4-Dihydroxyphenylacetic acid	102-32-9	C8H8O4	0.62	[24]
Protocatechuic acid	99-50-3	C7H6O4	17.61–965	[20,24]
Tyrosol	501-94-0	C8H10O2	3.33–168.12	[19,22,25]
Oleoside	178600-68-5	C16H22O11	46	[26]
Oleoside 11-methyl ester	60539-23-3	C17H24O11	23	[26]
**Secoiridoid derivatives**				
Oleuropein	32619-42-4	C25H32O13	2.81–5940	[16,17,18,19,20,21,22,23,25,26]
Secoxyloganin	58822-47-2	C17H24O11	1.80–138.32	[17]
Ligstroside	35897-92-8	C25H32O12	17	[26]
**Flavanols**				
Catechin	18829-70-4	C15H14O6	nd–77	[19,22,24]
Gallocatechin	970-73-0	C_15_H_14_O_7_	72	[22]
Epicatechin	490-46-0	C15H14O6	nd–2.17	[22,24,25]
Epicatechin gallate	1257-08-5	C22H18O10	133	[22]
Epigallocatechin	970-74-1	C15H14O7	3	[20]
**Flavones**				
Luteolin	491-70-3	C15H10O6	nd–266	[17,18,19,20,22,23,24]
Luteolin-7-o-glucoside	5373-11-5	C_21_H_20_O_11_	30–3978	[17,18,19,20,22,23,24,25]
Luteolin-4′-O-glucoside	6920-38-3	C_21_H_20_O_11_	3.97–330	[17,23]
Luteolin-3′,7-di-O-glucoside	52187-80-1	C_27_H_30_O_16_	6.37–31.38	[17]
Diosmetin-7-O-neohesperidoside	38665-01-9	C28H32O15	0.21–1.27	[17]
Apigenin	520-36-5	C15H10O5	nd–9.38	[17,19,22,24]
Apigenin-7-o-glucoside	578-74-5	C_21_H_20_O_10_	7.88–214.71	[17,19,20,22,24,25]
Apigenin-7-O-neohesperidoside	17306-46-6	C27H30O14	18.27–50.40	[17]
Hispidulin	1447-88-7	C16H12O	0.02–0.44	[17]
**Flavonols**				
Quercetin	117-39-5	C15H10O7	nd–22	[17,20,24]
Quercetin-3-o-glucoside	482-35-9	C21H20O12	6.48–31.65	[17]
Quercetin-3-o-galactoside	482-36-0	C21H20O12	1.08–3.06	[24,25]
Quercetin-4′-O-glucoside	20229-56-5	C21H20O12	nd–1.83	[17]
Rutin	153-18-4	C27H30O16	1.49–101	[17,19,20,22,25,26]
Kaempferol	520-18-3	C15H10O6	nd–1.18	[17,20,24,25]
Kaempferol-7-O-glucoside	16290-07-6	C21H20O11	62.19–268.11	[17]
Tiliroside	20316-62-5	C30H26O13	0.48–1.85	[17]
**Flavanonols**				
Taxifolin	480-18-2	C15H12O7	0.32–27.43	[17,24]
Taxifolin-3-glucoside	27297-45-6	C_21_H_22_O_12_	0.44–2.89	[17]
**Flavanones**				
Eriodictyol	552-58-9	C15H12O6	nd–44.77	[17,22,24]
Hesperidin	520-26-3	C28H34O15	nd–5.72	[22,24]
**Coumarins**				
Esculin	531-75-9	C15H16O9	0.07–2.09	[17]
Coumarin	91-64-5	C9H6O2	0.40–1.99	[17]
**Triterpenes**				
Asiatic acid	464-92-6	C30H48O5	0.88–2.88	[17]
Oleanonic acid	17990-42-0	C30H46O3	68.15–226.70	[17]
Maslinic acid	4373-41-5	C30H48O4	323.55–607.14	[17]
Corosolic acid	4547-24-4	C30H48O4	91.80–227.40	[17]
Oleanolic acid	508-02-1	C30H48O3	758.40–1047.67	[17]
Ursolic acid	77-52-1	C30H48O3	14.64–25.89	[17]
**Other compounds**				
Quinic acid	77-95-2	C7H12O6	605–2519	[18]
Pyrocatechol	120-80-9	C6H6O2	1.03	[24]
Pinoresinol	487-36-5	C20H22O6	nd–1.56	[24,25]

Range of content is expressed as milligrams per 100 g of dry weight. nd: no detectable.

## 3. Bioaccessibility and Bioavailability of Olive Leaf Polyphenols 

A crucial point of drug administration is the capacity of the active principle to be absorbed and passed to the systemic circulation, and to exert its action on the specific sites. In the case of a multicomplex food matrix such as the OL, it is necessary to evaluate the absorption and metabolism of numerous compounds present in it, and to evaluate the role of these compounds in the observed healthy effects. Some studies have investigated the pharmacokinetics of olive leaf phenolics by administering isolated compounds (not explored in this review). However, in this review, the bioaccessibility and bioavailability of individual compounds were examined, but after OL administration.

According with in vitro studies, gastric, intestinal, and colonic digestion significantly reduced the bioaccessibility of numerous compounds naturally present in OLs, such as phenolic acids (e.g., VB, chlorogenic, gallic, and caffeic acid), phenolic alcohols (e.g., HT and tyrosol), secoiridoid derivatives (e.g., OLE), flavones (e.g., luteolin 7-o-glucoside, apigenin 7-o-glucoside), flavanols (e.g., epicatechin), and flavonols (i.e., quercetin-3-o-rutinoside, quercetin-3-o-galactoside, kaempferol, and rutin) [25,26]. However, in vitro digestion also promoted the bioaccessibility and the potential bioavailability of some secoiridoid derivatives related to OLE hydrolysis, such as oleoside and oleoside 11-methyl ester [26].

To date, only three investigations have explored the bioavailability of phenolic compounds from OLs in humans. As can be observed in Table 2, numerous compounds such as secoiridoid derivatives, phenolic alcohols, and flavonoids can be found in plasma or urine samples after OL ingestion. No significant influence of gender on the absorption of OL phenolic compounds was observed in middle-aged people after OL consumption (270 or 400 mg/d). Interestingly, the administration of OLs through liquid glycerol preparation increased the plasma bioavailability of OLE and reduced the time to peak of HT derivatives compared with softgel capsule administration [27].

According to the pharmacokinetics, the biological half-life of plasma OLE metabolites (1.33–2.01 h) was shorter than that of HT derivatives (1.73–6.53 h), whereas the excretion peak rate in urine was 8-24 h for both metabolite classes [28,29]. The most abundant compounds found in urine were secoiridoids and HT and its derivatives, probably due to the rapid hydrolysis of OLE in the upper gastrointestinal tract [28,29]. It should be noted that the hydrolysis of OLE is not complete and numerous glucuronidated and sulfated derivatives from OLE can be found in plasma and urine, indicating that OLE is also conjugated by Phase II enzymes [29]. Curiously, a study of pre- and post-menopausal women fed with 250 mg of OL revealed that OL-related metabolites, such as HT glucuronide and sulfate, OLE aglycone glucuronide, and aglycon derivative I, were present in higher concentrations in the plasma from post-menopausal women. The authors attributed these results to potential age-related changes such as alterations in hormonal status and a decrease in gastric emptying [29]. These results are extremely interesting due the existence of a potential increase of bioavailability of phenolic compounds from OL, related, at least in part, to women’s age, opening the door to their potential use in aging and age-related diseases.

## 4. Toxicological Evaluation of Olive Leaves Bioactive Compounds

Olive leaves have been widely used as therapeutic tools throughout history [30]. In contrast to the classical belief that botanic-related products are completely safe and lack toxicity, these products could cause several side effects due to the fact that most of their chemical content remains uncharacterized. In addition, due to the easy access and low cost of these by-products, as well as the possibility of self-medication without medical advice for many people around the world, the study of OL-related toxicity is necessary. Therefore, in this section, the evidence regarding toxicity related to the intake of olive leaves is discussed.

According to in vitro studies, the co-incubation of different concentrations (51.2, 128, 320, 800, 2000, and 5000 µg/mL) of OL did not reveal pro-mutagenic effect in different *Salmonella typhimurium* (TA98, TA100, TA1535, and TA1537) and *Escherichia coli* (WP2 uvrA) strains in the Bacterial Reverse Mutation Test [31]. In the same way, coincubation with rising concentrations of OL (250, 500, 750, 1000, and 1250 µg/mL) did not affect the number of aberrant cells, polyploidy rates, or endoreduplication metaphases in V79 male Chinese hamster lung cells in the Chromosomal Aberration Test [31]. Similarly, treatment with lower OL dosages (20, 40, 60, 70, or 80 µg/mL) was demonstrated not to reduce or even increase viability in different cell lines [32,33].

Acute toxicity of OLs has also been evaluated in in vivo models. In this context, no adverse reactions, toxicity clinical signs, or lethality were observed after 24–48 h of OL administration in *Caenorhabditis elegans* (0.1, 1, 10, 100, 1000 µg/mL, [*C. elegans*]), NMRI BR mice (500, 1000, and 2000 mg/kg of body weight [bw]), or Swiss albino mice (2000 mg/kg bw) [31,34,35]. In fact, no genotoxic activity of OL was observed in bone marrow from these NMRI BR mice consuming 500, 1000, or 2000 mg/mL for 48 h [31] or *Drosophila melanogaster* acutely exposed to 3.75 or 30 µg/mL of OL [36]. Additionally, no embryolethality or embryotoxicity were found after an acute exposure to 100 µg/mL of OL for 24 h in *C. elegans* Wild-type strain [35]. Regarding sub-chronic toxicity of OL, the intake of 100, 200, 400, or 2000 mg/kg bw of OL daily via gavage for 14 or 28 days did not produce mortality, signs of toxicity, or behavioral and physical alterations in Wistar male and female rats. In addition, necropsy showed no abnormalities in the liver and kidney of treated rats [37]. Similar results were obtained in Wistar rats orally supplemented with 360, 600, or 1000 mg/kg bw of OL daily for three months [31]. Additionally, these authors found no alterations in organ weight (liver, adrenals, kidneys, thyroid/parathyroid, thymus, spleen, brain, heart, epididymides, testes, ovaries, fallopian tubes, and uterus) or pathological lesions in the most representative organs from locomotor, digestive, lymphatic, integumentary, respiratory, cardiovascular, endocrine, excretory, reproductive, and central and peripheral nervous systems [31]. Similarly, the consumption of 250 mg/day of OL in a double-blind, randomized controlled trial for 12 months revealed an absence of side effects in aged women [38]. In accordance with chronic toxicity, only one study evaluated the long-life effect of OL. In this context, lifelong administration of 100 µg/mL did not modify the survival rates in the *C. elegans* Wild-type strain [35].

Among the in vivo endpoint studies, some research has evaluated the influence of OL treatment in biochemical and hematological parameters. Interestingly, after an acute administration of 2000 mg/kg bw of OL, some hematological (hematocrit, hemoglobin, mean corpuscular volume, red blood cells, and platelets) and biochemical parameters (cholesterol and creatinine levels) were reduced without producing abnormalities in liver or kidneys [37]. It should be noted that the solvent used in this work was a solution made with 51% of ethanol, which could also interfere in hematological and biochemical studies, meaning results may not be attributed to the treatment itself. In fact, when the same solvent was administered for 28 days, the effect on hematological and biochemical parameters disappeared, probably due to an adaptation to alcohol consumption [37]. Similarly, the intake of 360, 600, or 1000 mg/kg bw of OL diluted in distilled water daily for three months did not alter most of the hematological parameters, electrolytes, or renal and hepatic markers studied in rats [31]. According to hepatic markers, no adverse effects were noted related to aspartate and alanine aminotransferase, gamma glutamyl transferase, and alkaline phosphatase levels in middle-aged people who consumed 270 or 400 mg of OL [27]. Similarly, no clinical changes were observed in classical biochemistry, hematological, or electrolytes parameters, or renal- and liver-function-related parameters, in a randomized controlled trial that administered 1000 mg/day of OL for 8 weeks [39].

## 5. Effects of Olive Leaf Bioactive Compounds in the Molecular Mechanisms Related to AD

In the following section, the scientific evidence regarding the effects of OL bioactive compounds on the main mechanisms involved in the pathogenesis of AD will be discussed. A summary can be found in Table 3.

### 5.1. Effects on Aβ Aggregation

As mentioned before, an abnormal extracellular accumulation and clearance of the Aβ in the brain is one of the main features of AD, which leads to neuron death and the typical symptoms of dementia [40]. In this regard, several studies have evidenced the protective role of OL and its bioactive molecules.

In vitro experiments indicated that individual compounds from OL were able to reduce both the aggregate size and occurrence of Aβ_42_ fibrils [40]. In the human neuroblastoma SH-SY5Y cell line, treatment with an OL fraction enriched in triterpenoid compounds (OLE, HT, VB, LU, and quercetin [QU]) reversed the loss of viability induced by the neurotoxic agent Aβ_1–42_. The lipid profile analysis performed using bioinformatic tools revealed that a significant number of phosphatidylcholines and phosphatidylethanolamines significantly increased, whereas several triacylglycerols decreased in the treated neuroblastoma cells [32]. In the same model, treatment with the aforementioned compounds and commercial preparation of OL exerted a stronger protection against Aβ42-, Cu-Aβ42-, or L-DOPA-Aβ42-induced neurotoxicity, manifesting an increase in cell viability [40]. In accordance with a computational binding affinity test, the neurotoxicity reduction mentioned above may be attributed to the ability of OLE, HT, LU, VB, and QUE, as well as their derivatives, to strongly bind to the hairpin-turn of the Aβ_1–40_ and Aβ_1–42_ monomers and the subsequent reduction in Aβ fibrillization [41]. On the other hand, β-secretase site-1 (BACE-1) is involved in the generation of the Aβ aggregation since it participates in the amyloidogenic processing of the amyloid precursor protein (APP). In vitro assays have demonstrated that bioactive compounds present in olive-tree-derived products, both non-flavonoids (e.g., HT, VB) and flavonoids (e.g., RU, QU), have a remarkable inhibitory effect on the BACE-1 enzyme. The commercial olive biophenol extracts (olive leaf extract rich in OLE or HT) also exerted a strong inhibitory activity, the latter being the most powerful. Although the action mechanism of extraction of olive biophenols is not clearly understood, the results showed a synergistic effect of the combination of flavonoid or non-flavonoid compounds in the extracts which are rich in biophenols [42].

Regarding the in vivo evidence, a transgenic strain of *C. elegans* expressing the human Aβ_1–42_ peptide in muscle cells was used by Romero-Márquez et al. (2022) for evaluating the anti-Aβ aggregation effect of an olive leaf extract containing 40% of OLE. Results showed a delay in the amyloid-induced paralysis of worms and a reduction in the amount of Aβ deposits stained by Thioflavin T. The RNAi test showed the participation of DAF-16/FOXO, SKN-1/Nrf2, and HSP16.2 pathways in those effects. This extract has been authorized to be used as an ingredient for nutritional supplements in human nutrition, so it could be a very promising approach for AD therapy [35]. Similar results were found for a HT-rich extract from olive fruit [43]. Apart from cells and nematode models, the anti-amyloid effect of olive leaves has also been evaluated in rodents. The AD models 5xFAD and APPswe/PS1dE9 mice overproduce the Aβ peptide and develop progressive cerebral Aβ deposits and learning and memory impairment. Olive leaf extract enriched in OLE mixed with the powdered food was able to reduce the total Aβ deposits in the hippocampus and cortex in both 5xFAD [44] and APPswe/PS1dE9 [40] animals. The soluble Aβ40 levels [44] and the size of Aβ plaques [40], respectively, were also reduced. Furthermore, an increase in the expression of Aβ clearance proteins (P-gp and LRP1) was observed in the treated group. The induction of anti-amyloidogenic protein and enzyme expression (sAPPα and α-secretase) and the reduction in the amyloidogenic protein sAPPβ suggested that the olive leaf extract is able to modulate APP processing [44]. The effect of an olive leaf extract containing 40% OLE on Aβ production was also investigated in male white rabbits, although not in an AD model but in one of cervical myopathy. The increase in Aβ in spinal cord tissue neuron cells after receiving compression treatment was effectively reduced by the treatment [45]. These results could be attributed to the high content of OLE aglycone present in olive leaves, which was also demonstrated to reduce Aβ-induced neurotoxicity through the reduction in Aβ aggregates in rats cerebrally injected with Aβ_42_ [46]. These results could be explained by results found by Brogi et al. (2020) using molecular docking. In this research, the authors showed that OLE aglycone was able to move deeply within the Aβ fibrils targeting a key motif in Aβ peptide, promoting structural instability and Aβ fibril disaggregation [47].

### 5.2. Molecules from Olive Leaves and NFTs Formation

Together with Aβ aggregation, AD is characterized by the intracellular accumulation of hyperphosphorylated NFTs. The Tau aggregation has been investigated using the experimental model *C. elegans*. In this case, a transgenic strain expressing the pro-aggregate human Tau protein in a constitutive pan-neuronal way was used. This strain manifests locomotion defects derived from Tau deposition. Treatment with an OL extract enriched in OLE (40%) improved several locomotive parameters related to Tau neurotoxicity through the modulation of the DAF-16/FOXO, SKN-1/Nrf2, and HSP16.2 pathways. Those transcription factors were also involved in the protective effect of the treatment against the Aβ proteotoxicity [35]. Likewise, in a rabbit model, extracts with the same percentage of OLE decreased the high levels of p-Tau in spinal cord tissue neuron cells after receiving compression treatment in the cervical myelopathy [45]. These results might be attributed to the high content of OLE, OLE aglycone, and HT, which have been shown to prevent Tau fibrillization in vitro [48].

### 5.3. Actions in Enzymes Related with Neurotransmitters Degradation

The action of specific enzymes on neurotransmitters or certain proteins can lead to the development and progression of neurodegenerative diseases such as AD. The cholinesterases (acetylcholinesterase [AchE] and butyrylcholinesterase [BchE]), histone deacetylase, and tyrosinase are some of the enzymes associated with AD. AChE and BChE hydrolyze choline esters degrade the neurotransmitter acetylcholine. In fact, one of the hypotheses of AD is the cholinergic hypothesis. This system is severely affected in AD, and the over-activation of the mentioned enzymes appears to promote amyloid Aβ fibril formation. The deficit in cerebral cholinergic transmitters ultimately results in memory loss with other cognitive symptoms that are characteristic of AD. In that sense, one enzyme involved is histone deacetylase, which is required for memory formation. Studies have shown the relation between defects in this enzyme and the development of neuropathology and Tau neurofibrillary tangles formation. Likewise, tyrosinase activity is related to processes and sequences involved in the progression of AD [42]. On the other hand, AChE and BChE were effectively inhibited by the aforementioned commercial leaf extracts rich in HT or OLE [42], and by an ethanolic extract of olive leaves from different geographic origins [49]. In addition, different types of olive leaf extracts obtained by supercritical fluid extraction also exerted that activity [50]. The best adsorbent was sea sand, which yielded extracts rich in triterpenes with moderate inhibitory activity of the enzyme [50]. In contrast, HT [42], OLE [42], or maslinic acid [51] alone were not able to inhibit the enzymes, whereas oleanolic acid [52,53,54] and pinoresinol [55] had a very slight inhibitory activity. In the same way, some representative compounds present in OL, such as tyrosol, luteolin 7-O glucoside, and ligstroside, showed a null correlation with both AChE and BchE inhibitory activity [56]. These results suggested that the enzyme inhibitory effect of OL might be caused, once again, by a synergism between different compounds present in the leaves.

### 5.4. Effects Related to Neuroinflammation

Inflammation is a common condition present in neurodegenerative diseases and is considered another pathological feature of AD. In vitro assays based on the ability of inhibiting the lipoxidase (LOX) demonstrated a modest anti-inflammatory potential of different types of OL extracts obtained by supercritical fluid extraction [50]. Positive results in that sense were also found in cell cultures. Treatment with green olive leaves containing OLE 20% in N1 murine microglia cell culture decreased BSA-AGE-induced NO production [33]. The LPS-induced increase of inflammatory markers was ameliorated by treatment with olive leaf extracts in human THP-1 monocytes [32] and in activated murine macrophages RAW 264.7 [57]. Concentrations of 20 and 40 µg/mL of an olive leaf fraction enriched in triterpenoid compounds reduced IL-6 and IL-1β secretion levels. Furthermore, the highest dose was able to reduce TNF-α levels in the monocyte cell line [32]. RAW 264.7 macrophages were treated with OLE-rich leaf extract in acute (50 µM extract with LPS for 24 h) or chronic exposure (50 µM extract pre-treatment for 24 h followed by LPS). Both acute and chronic treatment decreased NO production and strongly reduced the levels of iNOS and COX-2. In addition, both types also decreased the mRNA expression of IL-1β and IL-6, whereas the acute treatment was able to reduce IL-1βR protein expression and mRNA expression for TGF-β [57].

In the same line, the potential anti-inflammatory activity of olive leaves was also demonstrated in rodents. The 5xFAD mice model accumulated high levels of Aβ along with astrogliosis and microgliosis. Animals treated with an OL extract spiked in refined olive oil and mixed with powdered food showed less astrocyte activation and GFAP levels, together with an ameliorated astrocyte shape, compared with the control group fed only with the vehicle. Microglial activation in both the hippocampus and cortex was reduced, as were the IL-1β levels. In addition, the treatment also decreased NLRP3 in the brain, a finding which is associated with the significant reduction in pro-caspase-1 and pro-caspase-8. Components of the NF-κB, a classical pathway involved in inflammation, were also modulated by the extract. OL consumption significantly reduced the expression of p-IKKβ, an effect that was associated with increased levels of total IκBα and reduced p-IκBα [44]. In addition, the authors studied the receptor for advanced glycated end products (RAGE), which is considered a major source of Aβ entry to the brain and is related with the increase in pro-inflammatory cytokines. The interaction of RAGE with high mobility group box protein 1 (HMGB1) and the upregulation of this last protein in AD are well known. Protein levels of RAGE and HMGB1 were downregulated by the treatment. Overall, the mechanisms involved in the anti-inflammatory activity of olive leaves were the reduction in the NF-κB pathway, which regulates NLRP3 and RAGE/HGMB1 [44]. Likewise, the increase in TNF-α, IL-1β and prostaglandin E2 levels caused by lead (Pb) neurotoxicity were reduced with olive leaf extract in the hippocampus of male Wistar rats. Pb is a well-known neurotoxic agent considered to be a key mediator of inflammation and oxidative-stress-induced neuropathological effects. The oral administration of the extract was able to reduce tissue Pb deposition and prevent the negative effects [58]. However, oral treatment with olive leaf extract to kainic-acid-induced epilepsy Wistar rats did not exert a statistically significant decrease in the pro-inflammatory cytokine TNF-α, although other parameters related to oxidative stress were improved [59]. Additionally, treatment with OL extract containing 40% OLE resulted in a significant decrease in the inflammatory marker CD-68 (biomarker of activated microglia-macrophage) in a model of cervical myopathy in male white rabbits [45]. Inflammatory markers were also reduced in Peripheral Blood Mononuclear Cells (PBMCs) from male human patients treated for 8 weeks with 20 mL of liquid olive leaf extract, which provided 121.8 mg of OLE and 6.4 mg of HT daily. A downregulation of COX-2 and IL-8 gene expression was observed in PBMCs. Furthermore, the authors found a downregulation of the transcription factor jun-B, which is related to macrophage activation, and the Heparin binding EGF-like growth factor, both related to the NF-κB, in the treated group [60].

### 5.5. Oxidative Stress Attenuation

Oxidative stress (OS) is involved in the occurrence and progression of AD. Aβ plaques and NFTs elevation are associated with increased levels of oxidation products from proteins, lipids, and nucleic acids in the hippocampus and cortex [61]. In this context, OL supplementation was found to reduce DNA damage and protein carbonyls in human PBMCs [62,63,64]. At the brain level, several in vivo studies demonstrated that OL consumption reduced DNA fragmentation, protein carbonyls, lipid peroxidation, and peroxynitrite levels in different murine models of neurodegenerative diseases [58,59,65,66,67,68] or aging [69,70,71]. However, OL supplementation did not alter the urinary markers of oxidative status of healthy young adults, indicating a possible protective role of OL only in redox-homeostasis-impaired conditions such as aging or AD [72]. The antioxidant effects of OL may be explained by the capacity to reduce ROS or NOS content, which leads to the reduction in oxidizing molecules such as DNA, protein, and lipids from different tissues. In this context, some authors have demonstrated the role of OL as a ROS scavenger in vitro and in vivo. Among them, De Cicco et al. (2020) observed that the preincubation with OL was able to reduce the ROS content increase induced by sodium palmitate, a free radical generator, in RAW 264.7 cells [73]. More recently, Romero-Marquez et al. (2022) demonstrated that N2 Wild-type *C. elegans* strain supplemented with OL presented lower ROS content after an acute exposition to the prooxidant 2,2′-Azobis (2-methylpropionamidine) dihydrochloride [35]. Additionally, OL supplementation has demonstrated protective effects at the neuron level. In this context, a combination of OL with *Hibiscus sabdariffa* leaves (13:2, *w/w*) was able to reduce ROS content in human SH-SY5Y neuroblastoma cells damaged by H_2_O_2_ [74].

Beyond its free radical scavenger activity, OL supplementation has shown a modulatory activity over some inducible enzymes related to antioxidant response element (ARE). According to the literature, plasma glutathione levels and antioxidant enzyme activity, such as that of glutathione peroxidase (GSH-Px), catalase (CAT), and superoxide dismutase (SOD), which contribute to the progression of the disease, significantly decreased in early AD [75]. Among the neurodegenerative-like murine model studies, OL supplementation has been found to increase the brain activity of numerous AREs, such as SOD [65,66,67,68,69], CAT [66,67,68,69], GSH-Px [66,69], and glutathione S-transferase (GST) [58], as well as increase glutathione [67,76] brain levels. Notably, the common factor of these AREs is that they are regulated, totally or partially, by the nuclear factor erythroid 2-related factor 2 (Nrf2) [77]. Nrf2 is a transcription factor involved in the protection against OS. Under OS conditions, Nrf2 translocates to the nucleus and promotes the genetic expression of a significant number of AREs [78]. Clinically, the hippocampus from AD patients presents less nuclear Nrf2 compared to healthy controls, although OS markers are higher [79]. This feature indicates that AREs cannot be activated as Nrf2 does not translocate from the cytoplasm into the nucleus in hippocampal neurons in patients with AD [80]. Therefore, Nrf2 activation has been proposed as a novel target in the treatment of AD. Interestingly, some treatments, such as methylene blue, have demonstrated that the reduction in tauopathy, OS, and locomotive and memory impairment induced by NFT formation was mediated by Nrf2 activation in a mouse model of tauopathy [81]. In this context, some authors also described the capacity of OL to modulate Nrf2 in vitro [73] and in vivo [35,82]. Nonetheless, only one work researched the role of OL-induced Nrf2 nuclear translocation to fight AD in vivo. In this context, the authors focused on the role of OL in two different experimental *C. elegans* AD models. As mentioned in the previous sections, the authors demonstrated that OL treatment was able to reduce the cytotoxic effect of Aβ through the reduction in Aβ plaque aggregation. In the same way, the authors described a reduction in the neurotoxic effect caused by Tau aggregation in OL-treated animals. The authors partially described the mechanism of action of OL using RNAi technology, indicating a key role of SKN-1, a *C. elegans* ortholog of the human Nrf2, in the progression of Aβ and Tau protein cytotoxicity in *C. elegans* [35]. Therefore, the increase in Nrf2 translocation, and the subsequent activation of ARE, might be a possible mechanism of action underlying the protective anti-AD effect by olive leaf supplementation.

### 5.6. Autophagy and Proteostasis Modulation

Scientific evidence indicates that some AD hallmarks such as senile plaque formation are closely related to an alteration in the autophagic pathway and the incapacity to eliminate Aβ_1–42_ aggregates [83]. Indeed, alterations in autophagic-lysosomal degradation of proteins has been associated with AD, which were correlated with AD progression in both animal models and humans [83]. Recently, it has been demonstrated that the expression of MAPK/p38α protein is upregulated in the brain of APP-PS1 transgenic AD mouse, whereas the knockdown of MAPK in the APP-PS1 mouse stimulates macroautophagy/autophagy, reducing amyloid pathology by increasing autophagic-lysosomal degradation of BACE1 [84]. In the same way, high levels of phosphorylated AKT were associated clinically with the progression of NFT aggregation in AD patients [85]. These features seem to be consistent in a neurodegenerative rodent model induced by Pb. The exposure to Pb induced MAPK/p38 and AKT phosphorylation in the hippocampus of rats. Interestingly, OL supplementation was able to reduce both autophagy markers, which were associated with a reduction in Pb-induced neurotoxicity and an improvement of behavioral and locomotive tests [58].

Although there is limited data available about the modulatory effect of OL on autophagy markers during AD, Leri et al. (2021) investigated the role of an equal mix of OLE aglycone and HT in a cellular model of AD [86]. In this context, the Aβ_1−42_ oligomers’ exposure to human SH-SY5Y cells increased Beclin1, p62, and S6 expression, as well as the LC3II/I ratio. It seems that Aβ_1–42_ promotes the expression and activation of autophagy regulation markers, indicating an accumulation of autophagosomes with disrupted degradative activity [87]. In addition, p62 is remarkably involved in AD due to autophagic degradation through the binding of the autophagy marker LC3 [88]. With this background, HT-mix-treated cells presented a significant time-dependent reduction in p62 levels, which was reflected in lower Aβ_1–42_ oligomers on the surface, suggesting that these aggregates were digested by autophagolysosomes. In the same way, the phosphorylation level of the ribosomal protein S6, a key downstream substrate of TOR, was reduced in cells treated with the HT mix, indicating an involvement of the AMPK pathway in autophagy activation mediated by olive leaf phenols [86].

Similar to autophagy modulation, the role of proteostasis network modulation has been proposed as an intervention for AD management. There is extremely limited information about this topic. As far as it is known, the only work that evaluated the direct role of OL on the proteostasis network component during AD was evaluated in *C. elegans*. In this research, OL treatment was able to reduce both Aβ and Tau-protein-induced cytotoxicity, whereas an overexpression of HSP-16.2 was reported in a GFP-reporter strain. HSP-16.2 is an important element of protein homeostasis, which involves highly conserved stress responses that prevent protein mismanagement. In *C. elegans*, HSP-16.2 encodes HSP-16, which directly interacts with Aβ peptide and interferes with oligomerization pathways, leading to reduced formation of toxic species. To confirm the role of this protein in the protective effect shown by OL against AD, the authors used RNAi technology to knock down HSP-16.2 in two different AD-like strains of *C. elegans*. Interestingly, the authors demonstrated that the protective effect of OL to fight both Aβ and Tau-protein-induced cytotoxicity was mediated by HSP-16.2 overexpression. These results were confirmed by Thioflavin-T staining, which showed lower Aβ accumulation on OL-treated worms, probably due to an increase in Aβ clearance mediated by HSP-16.2 [35]. In conclusion, knowledge about the role of olive leaves in combating AD via autophagy/proteostasis modulation, in order to use it as AD prevention or therapy, is far from being complete. Nonetheless, the limited research available seems to indicate that the protective role of OL might be mediated by an enhancement of autophagy and proteostasis, although more research is needed.

**Table 3:** Mechanisms of action of OL in both in vitro and in vivo models.

Model	Intervention	Effects	Ref.
**Anti-Aβ aggregation**			
SH-SY5Y cells	Cells exposed to Aβ_42_ and treated with OL or QU (≥200 µM)	↓ Aβ_42_ fibrils aggregation	[40]
	Cells exposed to Aβ_42_ and treated with olive phytochemicals: OLE (IC_50_: 22.9 µM), HT (IC_50_: 30.4 µg/mL), OL (IC_50_: 45 µg/mL), OFE (IC_50_: 95.9 µg/mL), VB (IC_50_: 22.6 µM), LU (IC_50_: 36.9 µM) or QU (IC_50_: 45.9 µM)	↓ Aβ_42_ fibrils formation	
SH-SY5Y cells	Cells exposed to Aβ_1–42_ and treated with OL (40 µg/mL)	↑ Cells viability	[32]
SH-SY5Y cells	Cells pretreated with Olive phytochemicals: OLE, HT, VB, CA, LU, QU, RU or OFE (0–1000 µM) and injured with Aβ_42_, Aβ_42_ and Cu or Aβ_42_ and L-DOPA	↑ Cells viability	[40]
*C. elegans*	Animals exposed to OL (100 µg/mL) for 72 h	↓ Aβ-induced toxicity ↓ Aβ deposits	[35]
*C. elegans*	Animals exposed to OFE (100 µg/mL) for 72 h	↓ Aβ-induced toxicity ↓ Aβ deposits	[43]
5xFAD mice	Animals orally supplemented with OL (695 µg/kg/d) for 3 months	↓ Total Aβ deposits ↓ Soluble Aβ_40_ levels ↑ Expression of P-gp and LRP1 ↑ sAPPα and α-secretase expression ↓ sAPPβ expression ↑ Memory function	[44]
APPswe/PS1dE9 mice	Animals orally supplemented with OL (50 mg/kg/d) for 16 weeks	↓ Aβ plaques total number ↓ Aβ plaques size	[40]
New Zealand white rabbits	Animals spinally damaged and orally supplemented with OL (350 mg/kg/d) for 1 week after 14 days of spinal damage	↓ Aβ presence	[45]
	Animals spinally damaged and orally supplemented with OL (350 mg/kg/d) for 3 week after 7 days of spinal damage	↓ Aβ presence	
**Anti-tau aggregation**			
*C. elegans*	Animals exposed to OL (100 µg/mL) for 72 h	↑ Swimming speed and wavelength ↓ Stretching effort	[35]
New Zealand white rabbits	Animals spinally damaged and orally supplemented with OL (350 mg/kg/d) for 1 week after 14 days of spinal damage	↓ p-Tau presence	[45]
	Animals spinally damaged and orally supplemented with OL (350 mg/kg/d) for 3 week after 7 days of spinal damage	↓ p-Tau presence	
**Enzyme inhibitory activity**			
BACE-1, AChE, BChE, histone and tyrosinase inhibitor screening assay kits	Isolated olive phytochemicals: OFE (IC_50_: 18 ng), OLE (IC_50_: 2.7 µM) and HT (IC_50_: 0.26 µM)	↓ BACE-1 activity	[42]
	Isolated olive phytochemicals: OLE (IC_50_: 101.7 µM), and HT (IC_50_: 37.6 µM)	↓ AChE activity	
	Isolated olive phytochemicals: OLE (IC_50_: 84.8 µM) and HT (IC_50_: 30.6 µM)	↓ BChE activity	
	Isolated olive phytochemicals: HT (IC_50_: 66.22 µg), OLE (IC_50_: 1085 µM)	↓ histone deacetylase activity	
	Isolated olive phytochemicals: QU (IC_50_: 10.73 µM)	↓ Tyrosinase	
Microtitre assays	OL (500 µg/mL)	↓ AChE and BChE activity	[49]
Colorimetric assays	OL extracts from Cornicabra variety obtained with silicas (IC_50_: 300.80 to 805.47 µg/mL), aluminum oxide (IC_50_: 449.50 to 549.49 µg/mL), zeolites (IC_50_: 391.84 to 418.40 µg/mL) or adsorbates (IC_50_: 144.43 to 447.64 µg/mL)	↓ AChE activity	[50]
	OL extracts from Cornicabra variety obtained with sea sand adsorbate (IC_50_: 183.82 µg/mL)	↓ BChE activity	
**Anti-inflammation**			
Colorimetric assays	OL extracts from Cornicabra variety obtained with silicas (IC_50_: 83.53 to 548.87 µg/mL), aluminum oxide (IC_50_: 75.20 to 319.76 µg/mL), zeolites (IC_50_: 83.27 to 192.65 µg/mL) or adsorbates (IC_50_: 84.29 to 139.82 µg/mL)	↓ LOX activity	[50]
N1 murine microglia cells	Cells BSA-AGE injured and treated with green OL (EC_50_: 65 mg/mL)	↓ NO production	[33]
THP-1 cells	Cells injured with LPS and treated with OL (20 or 40 µg/mL)	↓ IL-1β, IL-6 secretion ↓ TNF-α secretion (only 40 µg/mL)	[32]
RAW 264.7 cells	Cells injured with LPS for 24 h (acute exposure) or 72 h (chronic exposure) and treated with OL (50 µM)	↓ NO, iNOS and COX-2 levels ↓ IL-1β and IL-6 mRNA expression ↓ IL-1βR protein expression and TGF-β mRNA expression (only in acute exposure)	[57]
5xFAD mice	Animals orally supplemented with OL (695 µg/kg/d) for 3 months	↓ Astrocyte and microglial activation ↓ GFAP levels ↓ Astrocyte shape alterations ↓ IL-1β levels ↓ NLRP3 levels ↓ Pro-caspase 1 and 8 levels ↓ RAGE and HMGB1 levels ↓ p-IKKβ and p-IκBα levels ↑ Total IκBα levels	[44]
Wistar rats	Animals neurologically damaged with Pb and orally supplemented with OL (0.1%) for 2 weeks	↓ TNF-α, IL-1β and PGE_2_ levels	[58]
Wistar rats	Animals neurologically damaged with kainic acid and orally supplemented with OL (300 mg/kg/d) for 4 weeks	=TNF-α	[59]
New Zealand white rabbits	Animals spinally damaged and orally supplemented with OL (350 mg/kg/d) for 1 week after 14 days of spinal damage	↓ CD68 levels	[45]
	Animals spinally damaged and orally supplemented with OL (350 mg/kg/d) for 3 week after 7 days of spinal damage	↓ CD68 levels	
Male human patients	Young people orally supplemented with 20 mL of liquid OL daily for 8 weeks	↓ COX-2, IL-8, JUNB, HBEGF gene expression in PBMCs	[60]
**Oxidative stress modulation**			
SH-SY5Y cells	Cells pretreated with OL (500 µg/mL) and exposed to H_2_O_2_ or Cu	↑ Cell viability	[89]
Colorimetric assays	OL extracts from Cornicabra variety obtained with Silicas (IC_50_: 46.43 to 1789.61 µg/mL), aluminum oxide (IC_50_: 39.99 to 191.74 µg/mL), zeolites (IC_50_: 128.49 to 153.55 µg/mL), and adsorbates (IC_50_: 23.65 to 148.22 µg/mL)	↓ ABTS	[50]
	OL extracts from Cornicabra variety obtained with sea sand adsorbate (IC_50_: 18.27 µg/mL)	↓ ROS	
	OL extracts from Cornicabra variety obtained with sea sand adsorbate (IC_50_: 1036.86 µg/mL)	↓ RNS	
SH-SY5Y cells	Cells exposed to a mix (13:2) of OL and *Hibiscus sabdariffa* L. Flowers (50, 100, 250, 500, 1000 µg/mL)	↓ ROS content	[90]
SH-SY5Y cells	Cells exposed to H_2_O_2_ and treated with a mix (13:2) of OL and *Hibiscus sabdariffa* L. Flowers (0–50 µg/mL)	↓ ROS content	[74]
SH-SY5Y cells	Cells exposed to Aβ_1–42_ and treated with a mix (75 µM) based on OLE and HT	↓ ROS content	[86]
*C. elegans*	Animals treated with OL (100 µg/mL) for 72 h and exposed to AAPH	↓ ROS content	[35]
Wistar rats	Aged animals orally supplemented with OL (50 mg/kg/d) for 6 months	↑ Midbrain SOD, GPX and CAT activities ↓ Midbrain MDA levels	[69]
Wistar rats	Animals neurologically damaged with Pb and orally supplemented with OL (0.1%) for 2 weeks	↓ Frontal cortex DNA fragmentation ↑ GST activity	[58]
Wistar rats	Aged animals orally supplemented with OL (500 and 1000 mg/kg/d) in drinking water for 2 months	↓ Brain MDA, diene conjugate and protein carbonyl (highest dose)	[70]
Mongolian gerbils	Animals pretreated with OL (100 mg/kg/d) and subjected to global cerebral ischemia	↓ Superoxide and nitric oxide production ↓ Lipid peroxidation ↑ SOD activity	[65]
Wistar rats	Animals pretreated with OL (75, 150 and 300 mg/kg/d) for 28 days and neurologically damaged with rotenone	↓ Midbrain MDA levels ↑ Midbrain SOD, CAT, and GPx levels	[66]
Wistar rats	Animals neurologically damaged with kainic acid and orally supplemented with OL (300 mg/kg) for 4 weeks	↓ The seizure score ↓ Neuron MDA, nitrite, and nitrate levels ↑ GSH levels	[59]
**Autophagy and proteostasis modulation**			
SH-SY5Y cells	Cells exposed to Aβ_1–42_ and treated with a mix (75 µM) based on OLE and HT	↑ Autophagic flux ↑ p62 levels	[86]
*C. elegans*	Animals exposed to OL (100 µg/mL) for 72 h	↑ HSP-16.2::GFP expression	[35]

Aβ: amyloid beta; AChE: acetylcholine esterase; AGE: advanced glycated end products; BACE-1: prime Aβ producing enzyme β-secretase; BChE: butyrylcholine esterase; BSA: bovine serum albumin; *C. elegans*: *Caenorhabditis elegans*; CD68: cluster of differentiation 68; COX-2: cyclooxygenase-2; EC50: half maximal effective concentration; GFAP: glial fibrillary acidic protein; HBEGF: Heparin binding EGF-like growth factor; IC50: half maximal inhibitory concentration; IL: interleukin; HT: hydroxytyrosol; L-DOPA: levodopa; LPS: lipopolysaccharide; LOX: lipoxydase; NO: nitric oxide; OL: olive leaves; OFE: olive fruit extracts; OLE: oleuropein; QU: quercetin; RU: rutin; CA: caffeic acid; LU: luteolin; VB: verbascoside; PBMCs: peripheral blood mononuclear cells; PGE2: prostaglandin E2; RAGE: receptor for advanced glycated end products; TGF-β: transforming growth factor β; TNF-α: tumor necrosis factor α; SOD: superoxide dismutase; CAT: catalase; MDA: Malondialdehyde; GPX: glutathione peroxidase; GST: Glutathione-S transferase; MAPK: p38 mitogen activated protein kinase; TRAP: total radical trapping antioxidant parameter; ROS: reactive oxygen species; RNS: reactive nitrogen species. The symbols ↑ and ↓ mean an increase or decrease of the specific marker, respectively.

## 6. Trends, Perspectives, and Conclusions

**Figure 2:**
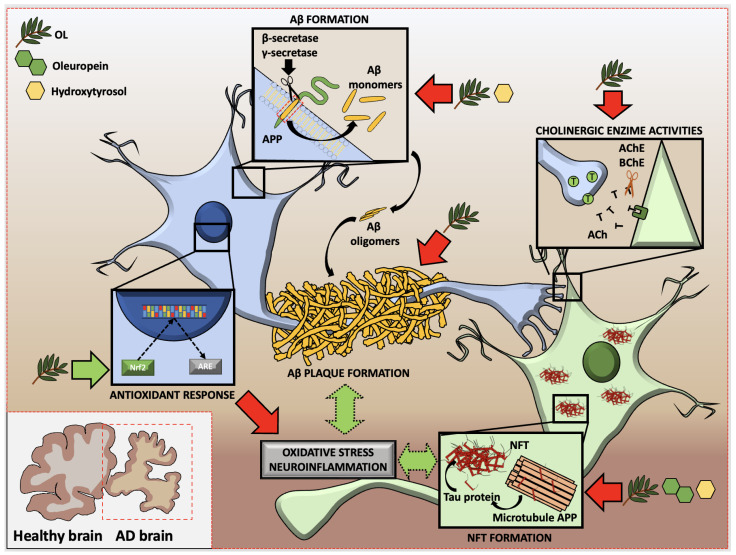
Modulatory effect of olive phytochemicals on Alzheimer’s Disease physiopathology. Green arrows represent promotion of the particular process. Red arrows represent reduction in the particular process. Green discontinued arrows represent indirect promotion of the particular process. AChE: acetylcholinesterase; AD: Alzheimer’s disease; APP: amyloid precursor protein; Aβ: amyloid beta; BChE: butyrylcholinesterase; NFT: neurofibrillary tangles; OL: olive leaf.

Despite all the efforts, AD continues to remain a challenge, with no effective treatment to combat it, increasing the dependency and, subsequently, the death, of patients. Natural products such as olive leaves and their compounds contribute to the discovery of new anti-AD interventions to combat AD progression. The effectiveness of OL, and of the molecules present in this olive tree by-product, in reducing or even preventing the different processes related to AD, including Aβ and Tau protein production, Aβ fibrogenesis and NFT deposition, inhibition of AD-related enzymes, and oxidative stress and neuroinflammation, have been described (Figure 2). In particular, OL, OLE, and HT reduced both amyloid-β formation and neurofibrillary tangles formation through amyloid protein precursor processing modulation. Although the isolated olive phytochemicals exerted lower cholinesterase inhibitory activity, OL demonstrated high inhibitory activity in the cholinergic tests evaluated. The mechanisms underlying these protective effects might be associated with decreased neuroinflammation and oxidative stress via NF-κB and Nrf2 modulation, respectively.

Furthermore, memory impairment is associated with early AD stage. However, with AD progression, patients tend to develop symptoms of cognitive and behavioral alterations, such as depression, anxiety, disorientation, and paranoia, which affect daily living activities [91]. Some authors have explored the potential therapeutic effect of OL consumption to combat the deleterious effect of neurodegenerative disease induced by chemicals on memory and cognitive function. OL consumption was able to restore the locomotive impairment caused by Pb-induced neurological damage in rats subjected to an open field test [58]. Similarly, in a rat model of Parkinson’s disease induced by rotenone, OL consumption was able to partially restore balance, motor coordination, and muscle strength in a dose-dependent manner [66]. The limited research available that addresses the therapeutic effect of OL in AD-like symptoms failed to find a significant test to measure the behavioral alterations related to AD progression. In this context, Omar et al. (2019) tried to evaluate some behavioral analysis related to anxiety, locomotive function, and orientation in APPswe/PS1dE9 and Wild type mice supplemented or not with OL. As mentioned previously, APPswe/PS1dE9 mice overproduce human Aβ, causing learning and memory impairment. After 5 months of housing, the applied tests failed to show impairments in the studied behavioral parameters in AD or Wild type mice, although Aβ plaque senile deposits were found in AD mice. These results indicate that the test, the age of the mice, or the AD phenotype used were not appropriate for the parameters studied. As mentioned above, behavioral parameters such as anxiety, orientation, and locomotive function are linked to late stages of AD, and it is necessary to produce a relatable late AD stage model with measurable results. In contrast to behavioral tests, Abdallah et al. (2022) found an improvement in memory function using the Morris water maze in OL-treated AD-like 5xFAD mice. Despite the promising results obtained in the Morris water maze test, the authors also failed to measure locomotive impairment related to AD, finding no differences between Wild type and AD mice, treated or not [40]. Although these results suggest that memory impairment related to AD progression might be restored by OL supplementation, more research is necessary to find a correct test to analyze AD behavioral impairment.

Summarizing, despite the fact that direct evidence is still limited, many investigations corroborate the potential use of OL as an adjuvant in AD treatment. Olive leaves have been proven to reduce the toxicity of protein aggregation through the reduction in Aβ formation and aggregation, as well as the reduction in Tau fibrogenesis and deposition. In the same way, the possible mechanism of action underlying the protective effect might be attributed to oxidative stress and neuroinflammation modulation, as well as an increase in toxic protein clearance through the modulation of autophagy and the proteostasis network. Nonetheless, most of the reviewed evidence comes from in vitro studies, indicating that more preclinical and clinical research is needed for a deeper understanding of the molecular mechanisms associated with the observed effects.

## Figures and Tables

**Table 2:** OL related metabolites found in plasma and urine samples in humans.

Phenolic Compound	Molecular Formula	Plasma	Urine	Refs.
**Phenolic alcohols**				
Hydroxytyrosol	C_8_H_10_O_3_	-	✓	[28]
Hydroxytyrosol sulfo glucuronide	C_14_H_18_O_12_	✓	✓	[27,29]
Hydroxytyrosol glucuronide	C_14_H_18_O_9_	✓	✓	[27,29]
Hydroxytyrosol sulfate	C_8_H_10_O_6_	✓	✓	[27,29]
Hydroxytyrosol-acetate glucuronide	C_16_H_20_O_10_	✓	✓	[29]
Tyrosol	C_8_H_10_O_2_	-	✓	[27]
Tyrosol glucuronide	C_14_H_18_O_8_	✓	✓	[29]
Homovanillic alcohol	C_9_H_12_O_3_	-	✓	[28]
Homovanillic alcohol sulfate	C_9_H_12_O_6_S	X	✓	[29]
Homovanillic alcohol glucuronide	C_15_H_20_O_9_	✓	✓	[29]
**Secoiridoid derivatives**				
Oleuropein	C_25_H_32_O_13_	✓	✓	[27]
Oleuropein aglycone	C_19_H_22_O_8_	-	✓	[28]
Oleuropein aglycone dialdehyde	C_17_H_20_O_6_	-	✓	[28]
Oleuropein aglycone derivative 1	C_25_H_32_O_15_	✓	✓	[29]
Oleuropein aglycone derivative 2	C_25_H_32_O_14_	✓	✓	[29]
Oleuropein aglycone glucuronide	C_25_H_30_O_14_	✓	✓	[29]
Oleuropein sulfate	C_19_H_22_O_11_S	✓	✓	[29]
Elenolic acid	C_11_H_14_O_6_	X	✓	[29]
Elenolic acid glucuronide	C_17_H_22_O_12_	X	✓	[29]
**Flavones**				
Luteolin	C_15_H_10_O_6_	✓	X	[29]
Luteolin glucuronide	C_21_H_18_O_12_	✓	X	[29]

✓: indicates presence of the compound in the sample; X: indicates the absence of the compound in the sample; -: indicates the absence of evidence about the specific metabolite in the sample.
